# Data-driven reweighting of the Charlson and Elixhauser comorbidity indices using German claims data: development for in-hospital mortality only, external validation across clinical and health-economic outcomes

**DOI:** 10.1186/s13561-026-00888-9

**Published:** 2026-10-27

**Authors:** Klaus Kaier, Derek Hazard, Adrian Heidenreich, Alexander Maier, Dirk Westermann, Constantin von zur Mühlen, Vera Oettinger

**Affiliations:** 1 Institute for Medical Biometry and Statistics, Faculty of Medicine and Medical Center, University of Freiburg, Stefan-Meier-Str. 26, Freiburg, 79104 Germany; 2 Center for Big Data Analysis in Cardiology (CeBAC), Department of Cardiology and Angiology, Medical Center, Faculty of Medicine, University Heart Center, University of Freiburg, University of Freiburg, Freiburg, Germany; 3 Department of Cardiology and Angiology, Faculty of Medicine, University Heart Center, Medical Center University of Freiburg, University of Freiburg, Freiburg, Germany

**Keywords:** Comorbidity index, Charlson, Elixhauser, Adaptive lasso, Health economics, Administrative data, Risk adjustment, Mortality, Bleeding, Mechanical ventilation, Length of stay, Reimbursement

## Abstract

**Background:**

Comorbidity scores such as the Charlson and Elixhauser indices are widely used for risk adjustment in health-economic and clinical outcomes research. Yet the existing weighting schemes, originally designed for mortality prediction, may no longer reflect today’s patient populations or perform equally well across diverse outcomes. This study aimed to reweight both indices using a data-driven approach and to evaluate their predictive validity for multiple clinical and health-economic outcomes.

**Methods:**

Using administrative data from a large tertiary care teaching hospital in Germany (*N* = 198,819 hospitalizations), we applied adaptive lasso regression to recalibrate the Charlson (Deyo weights) and Elixhauser (van Walraven weights) comorbidity indices. Reweighting was performed for in-hospital mortality as the development endpoint. Penalized coefficients were converted into integer-based weights. Model performance was assessed for three dichotomous endpoints (in-hospital mortality, severe bleeding, prolonged mechanical ventilation) and two continuous outcomes (length of hospital stay, total reimbursement). External validation was performed in three independent cohorts: Elective endometrial cancer (*N* = 85,204), interventional left atrial appendage closure (*N* = 28,039), and emergency ST-elevation myocardial infarction (STEMI; *N* = 248,062). AUC, R², and RMSE were used as complementary measures of discrimination and predictive accuracy. Pooled relative improvements of AUC, R², and RMSE across the three validation cohorts was calculated using random effects meta-analyses.

**Results:**

Reweighting consistently improved predictive performance across endpoints and cohorts, with modest absolute gains for in-hospital mortality and more pronounced gains for severe bleeding and continuous outcomes. When analysing in-hospital mortality in the three validation cohorts, the reweighted Charlson improved AUC from 0.679 to 0.714 in left atrial appendage closure, 0.588 to 0.594 in STEMI, and 0.798 to 0.810 in endometrial cancer (pooled relative increase: 2.05% [95% CI 0.56–3.53]); the reweighted Elixhauser showed larger gains, particularly in STEMI (0.708 to 0.766) and left atrial appendage closure (0.780 to 0.818; pooled relative increase: 4.86% [0.07–9.65]). The Elixhauser reweighting demonstrated consistently larger gains for severe bleeding (pooled AUC increase: 6.57% [4.23–8.92]). For continuous outcomes, the reweighted Elixhauser index yielded the largest gains in explained variance, with a pooled relative R² increase of 39.66% [33.72–45.60] for total reimbursement. Corresponding reductions in RMSE were modest and often not statistically significant.

**Conclusions:**

Adaptive lasso reweighting consistently enhances the predictive validity of the Charlson and Elixhauser indices across both clinical and health-economic endpoints. Our multi-endpoint evaluation shows consistent performance gains across the three examined German validation cohorts, offering a practical basis for risk adjustment in health-economic studies using German claims data. Because development and validation were based exclusively on German data and a single coding system (ICD-10-GM), international validation across other coding systems and healthcare systems is required before broader applicability can be assumed.

**Supplementary Information:**

The online version contains supplementary material available at https://doi.org/10.1186/s13561-026-00888-9.

## Introduction

Accurate adjustment for comorbidities is essential for valid inference in observational health-services and health-economic research. In administrative or claims-based data, the Charlson Comorbidity Index and the Elixhauser Comorbidity Index are the most frequently used tools to quantify patients’ morbidity burden and control for baseline risk differences in analyses of in-hospital mortality, complications, and healthcare costs [1, 2]. The Charlson index, first developed to predict one-year mortality, includes 17 chronic conditions and has since been adapted for use in hospital discharge datasets, most notably in the version by Deyo et al. (1992) [3]. The Elixhauser system, in contrast, was designed from the outset for administrative data and includes a broader range of acute and chronic diseases. Both indices have been recalibrated and reweighted repeatedly to improve their predictive accuracy in different settings [4–8].

Despite their widespread use, several limitations of traditional weighting systems remain. The original Charlson and Elixhauser weights were optimized primarily for mortality prediction. They may not capture risk heterogeneity relevant to other clinically or economically meaningful outcomes such as length of stay or reimbursement. Moreover, evolving disease prevalence, changing coding practices, and advances in care delivery may have altered the relationships between comorbidities and adverse outcomes since the indices were first developed. Recent studies have shown that re-estimating comorbidity weights with contemporary data can improve predictive validity. Sharma et al. (2021) demonstrated that newly derived Swiss Elixhauser weights achieved slightly higher discrimination for in-hospital mortality than the van Walraven system [8], while Kaier et al. (2024) showed that adaptive lasso reweighting of the Hospital Frailty Risk Score substantially enhanced discrimination and calibration in both local and national validation cohorts [9].

Building on this evidence, the present study applies adaptive lasso regression to derive new data-driven weights for both the Deyo- and van Walraven-based indices using routine inpatient data from a large tertiary care teaching hospital in Germany. Beyond assessing discrimination for in-hospital mortality, this study expands evaluation to continuous health-economic outcomes (length of stay and total reimbursement) to comprehensively test model performance in terms of discrimination (area under the receiver operating characteristic curve, AUC), explained variance (R²), and prediction error (root mean square error, RMSE). External validity is assessed in three independent datasets representing distinct clinical populations: elective endometrial cancer, interventional left atrial appendage closure, and emergency ST-elevation myocardial infarction (STEMI). Combining binary and continuous endpoints across diverse patient groups allows us to examine how well the reweighted indices generalize across the three clinical settings examined here.

## Methods

Similar to the approach described by Deyo et al. (1992) [3], we used multiple cohorts for model development and validation.

The development cohort contains data of all patients hospitalized between 2012 and 2015 at the Medical Center – University of Freiburg, which is a tertiary care teaching hospital in southern Germany. The development cohort was intentionally chosen to be broad and heterogeneous. Since all patients of a maximum care provider over several years are included, it can be expected that the full range of inpatients in Germany is considered. The dataset comprised patient characteristics including sex, age, primary and secondary diagnoses as well as in-hospital mortality. The unit of analysis was the individual hospitalization. Repeat admissions by the same patient within the observation period were not excluded, as is standard in administrative-data analyses where comorbidity scoring is applied at the level of each discrete episode of care.

Coding of diagnoses is based on the ICD-10 German Modification and Quan et al.’s (2005) [7] ICD-10 codes were used to determine each of the 17 Charlson and 31 Elixhauser comorbidities.

The development of reweighted scores was performed in two steps. First, we used variable selection to identify the relevant variables for the relationship with the analysed outcomes. Variable selection was performed using adaptive lasso [10], implemented via the Stata command “lasso logit” (binary logistic regression). The regularization parameter λ was selected by 10-fold cross-validation minimizing deviance. Adaptive weights for each predictor were constructed as the inverse of the absolute value of coefficients from an initial unpenalized logistic regression, applying lighter penalization to stronger predictors and heavier penalization to weaker ones. All predictors are binary indicators and were not standardized prior to estimation. Penalized coefficients from the adaptive lasso model were used directly for weight derivation without post-selection refitting. Compared with standard lasso [11], the adaptive lasso possesses the oracle property: asymptotically, it selects exactly the truly relevant predictors and assigns zero coefficients to irrelevant ones [10].

Afterwards, we used the model’s penalized coefficients to get weights for each dichotomous condition. The weighting rule developed by Schneeweiss et al. 2003 was applied to the penalized coefficients of the logistic regression model to obtain weights for each dichotomous condition [4]. Specifically, the estimated logistic regression coefficient was divided by 0.30 and rounded to the nearest integer. Thus, a weight of 1 corresponds to an exp(0.30) = 35% increase in the odds of in-hospital death [12]. The reweighted comorbidity scores were derived exclusively using in-hospital mortality as the development endpoint. Subsequent analyses of severe bleeding, prolonged mechanical ventilation, length of hospital stay, and total reimbursement represent validations of these mortality-derived weights across additional endpoints, not endpoint-specific reweighting.

The model performance is evaluated on the basis of the AUC. 95% confidence intervals (95% CI) and tests between two AUC are conducted with the nonparametric DeLong test [13].

Three different datasets were used as validation cohorts. These datasets were originally collected to answer disease-specific questions such as treatment effects. Each of these datasets was formed in collaboration with a clinician from the respective specialty and using ICD and OPS coding as inclusion and exclusion criteria. In detail, the first validation cohort comprised patients undergoing catheter-based left atrial appendage closure between 2016 and 2020, representing planned interventional cardiology procedures in patients with atrial fibrillation [14]. The second cohort included patients hospitalized with ST-elevation myocardial infarction (STEMI) between 2017 and 2020, thus representing emergency cardiology cases with a clearly defined main diagnosis [15]. The third dataset consisted of women undergoing surgery for endometrial cancer between 2007 and 2018, representing elective gynecologic oncology procedures with a predominantly non-cardiological patient population [16]. Together, these datasets provide complementary settings to externally validate the reweighted comorbidity scores across distinct clinical and procedural contexts.

In all validation cohorts, in-hospital mortality served as the primary endpoint. In addition, severe bleeding (transfusion of more than five red blood cell concentrates) and prolonged mechanical ventilation (> 48 h) were analyzed as dichotomous secondary endpoints to assess the ability of the scores to predict relevant non-lethal complications [17–19]. Both outcomes represent clinically meaningful and resource-intensive adverse events that closely reflect patients’ comorbidity burden and physiological resilience and are analyzed using AUC assessment and the nonparametric DeLong test. Severe bleeding, often related to anticoagulation, frailty, or organ dysfunction, is a frequent and prognostically relevant complication across medical and surgical settings. Similarly, prolonged mechanical ventilation is a robust indicator of postoperative or postprocedural morbidity, capturing respiratory failure and overall clinical deterioration. Including these outcomes enables evaluation of the comorbidity scores beyond mortality, encompassing major morbidity and healthcare resource use.

In addition, the continuous outcomes length of hospital stay and total reimbursement were analyzed using linear regression analyses in all validation cohorts. Predictive performance was quantified using the coefficient of determination (R²) and the RMSE. R² assessed the proportion of variance explained by the scores, while RMSE provided a measure of absolute predictive accuracy. To ensure robustness, 95% confidence intervals for both metrics were obtained using bootstrapping with 50 replications.

Based on the results of the DeLong test in the validation cohorts, random effects meta-analyses according to the DerSimonian-Laird model are conducted to outline the relative AUC-increase across the different validation datasets. Given the small number of pooled datasets (*n* = 3), the DerSimonian-Laird estimates of between-cohort heterogeneity are inherently imprecise, and the pooled results should be interpreted as an exploratory weighted summary rather than a definitive pooled effect estimate. For the continuous outcomes, standard errors from the bootstrapping approaches are used to quantify relative R²-increases and RMSE-decreases across the validation cohorts using random effects meta-analyses. Calibration plots were constructed by plotting observed mortality against model-predicted mortality in the three validation cohorts using fractional-polynomial prediction plots.

Regarding the development cohort, researchers were provided with only anonymized data in accordance with German data protection law. Therefore, patient consent was not required. The collection and use of patient data has been approved by the institutional ethics committee of the University of Freiburg (Research Ethics Committee of the Albert-Ludwigs-Universität Freiburg, Germany; decision number 305/14 on June 30th, 2014). Regarding the validation cohorts, Destatis granted access only to summarized results and the investigators did not have direct access to individual patient data. Therefore, approval by the ethics committee and informed consent were not required according to German law. To ensure privacy, Destatis anonymized all pooled results and all information that could identify an individual patient or center was censored by Destatis. This study has been performed in accordance with the Declaration of Helsinki.

The analyses were performed using Stata 17 (StataCorp, College Station, Texas, USA). Analyses are descriptive, p-values from the DeLong test are reported for the primary endpoint (in-hospital mortality) only without adjustment for multiple testing.

## Results

The study population comprised 198,819 hospitalizations at the tertiary care teaching hospital used for score development. In-hospital mortality was 2.3%. Baseline age, sex, and comorbidity patterns are summarized in Table 1; these data also report the prevalence of individual Charlson and Elixhauser conditions in the development cohort. In keeping with the pre-specified adaptive lasso approach, several low-informative comorbidities were excluded and penalized coefficients were transformed into integer weights as described in the Methods. For the Charlson reweighting, 4 of 17 conditions were removed and 13 retained, with the strongest positive association observed for moderate/severe liver disease (odds ratio [OR] 4.78; reweighted weight = 5). For the Elixhauser reweighting, 10 of 31 conditions were removed; among retained predictors, examples of positively weighted conditions included coagulopathy (OR 3.86; weight = 5) and fluid/electrolyte disorders (OR 3.37; weight = 4), while several conditions received negative weights consistent with lower mortality risk (e.g., uncomplicated arterial hypertension weight = − 1). Details for all conditions and weights are shown in Table 1 and Table S1.

**Table 1:** Score development in the Freiburg cohort (all patients hospitalized between 2012 and 2015 in Freiburg, *N* = 198,819)

Charlson Comorbidities	Charlson score					Elixhauser score			
	Mean	Weight	Coefficient	Odds Ratio	reweighted	Weight	Coefficient	Odds Ratio	reweighted
Myocardial infarction	3.53%	1	0.51	1.67	2				
Congestive heart failure	3.85%	1	1.07	2.91	4	7	0.57	1.76	2
Peripheral vascular disease	6.70%	1	0.31	1.37	1	2	0.28	1.33	1
Cerebrovascular disease	8.06%	1	1.29	3.65	4				
Dementia	1.36%	1	0.50	1.65	2				
Chronic pulmonary disease	7.67%	1	0.21	1.23	1	3	0.13	1.14	0
Rheumatologic disease	0.56%	1							
Peptic ulcer disease	0.54%	1	1.26	3.53	4	0	0.61	1.84	2
Mild liver disease	3.25%	1	0.51	1.67	2	11	0.36	1.43	1
Diabetes without chronic complications	10.41%	1				0	0.05	1.06	0
Diabetes with chronic complications	2.17%	2	-0.12	0.88	0	0			
Hemiplegia or paraplegia	4.84%	2	0.65	1.92	2	7	0.79	2.19	3
Renal disease	8.65%	2	0.54	1.71	2	5	0.22	1.25	1
Any malignancy	21.77%	2	0.45	1.57	2				
Moderate or severe liver disease	0.90%	3	1.56	4.78	5				
Metastatic solid tumor	10.36%	6	1.18	3.25	4	12	0.87	2.38	3
AIDS/HIV	0.25%	6				0			
Additional Elixhauser Comorbidities									
Cardiac arrhythmias	12.09%					5	0.83	2.30	3
Valvular disease	2.75%					-1			
Pulmonary circulation disorders	1.92%					4	0.67	1.96	2
Hypertension, uncomplicated	31.65%					0	-0.39	0.68	-1
Hypertension, complicated	1.35%					0	-0.73	0.48	-2
Other neurological disorders	7.49%					6	0.78	2.19	3
Hypothyroidism	7.43%					0	-0.31	0.73	-1
Lymphoma	2.68%					9	-0.14	0.87	0
Solid tumor without metastasis	18.13%					4	0.24	1.28	1
Rheumatoid arthritis	2.10%					0	-0.14	0.87	0
Coagulopathy	8.22%					3	1.35	3.86	5
Obesity	6.33%					-4	-0.36	0.70	-1
Weight loss	2.15%					6	0.93	2.55	3
Fluid and electrolyte disorders	11.16%					5	1.22	3.37	4
Blood loss anemia	0.71%					-2			
Deficiency anemia	1.54%					-2	-0.29	0.75	-1
Alcohol abuse	3.38%					0			
Drug abuse	5.45%					-7	-0.26	0.77	-1
Psychoses	0.46%					0			
Depression	5.43%					-3	-0.17	0.85	-1

Discrimination for in-hospital mortality improved consistently with the reweighted scores across the development and all external validation cohorts (Table 2). In the development cohort (all patients in Freiburg, *N* = 198,819), AUC (95% CI) increased from 0.759 [0.752–0.766] with the original Charlson to 0.786 [0.780–0.793] with the reweighted Charlson, and from 0.832 [0.826–0.837] with the original Elixhauser to 0.865 [0.860–0.871] with the reweighted Elixhauser (both *p* < 0.001). In elective endometrial cancer (*N* = 85,204), AUC were 0.798 → 0.810 (Charlson, *p* = 0.032) and 0.860 → 0.873 (Elixhauser, *p* = 0.005); in interventional left atrial appendage closure (*N* = 28,039), 0.679 → 0.714 and 0.780 → 0.818 (both *p* < 0.001); in emergency STEMI (*N* = 248,062), 0.588 → 0.594 and 0.708 → 0.766 (both *p* < 0.001), respectively. These cohort-specific estimates, rather than the pooled summaries reported below, form the primary basis for assessing the performance of the reweighted indices.

**Table 2:** Model discrimination regarding the endpoint in-hospital mortality

	Development cohort	External validation cohorts		
	All patients in Freiburg	Elective endometrial cancer	Interventional left atrial appendage closure	Emergency STEMI
	(*N* = 198,819)	(*N* = 85,204)	(*N* = 28,039)	(*N* = 248,062)
	AUC [95% CI]	AUC [95% CI]	AUC [95% CI]	AUC [95% CI]
Charlson score	0.759 [0.752–0.766]	0.798 [0.780–0.816]	0.679 [0.653–0.705]	0.588 [0.584–0.591]
Charlson score - reweighted	0.786 [0.780–0.793]	0.810 [0.791–0.828]	0.714 [0.687–0.740]	0.594 [0.590–0.597]
Elixhauser score	0.832 [0.826–0.837]	0.860 [0.845–0.875]	0.780 [0.756–0.804]	0.708 [0.704–0.711]
Elixhauser score - reweighted	0.865 [0.860–0.871]	0.873 [0.858–0.887]	0.818 [0.795–0.841]	0.766 [0.763–0.769]

Calibration plots for in-hospital mortality in the three validation cohorts are provided in Supplementary Figure S1, and quantitative calibration metrics for all indices and cohorts, comprising calibration-in-the-large, the optimism-corrected calibration slope and the integrated calibration index, are reported in Supplementary Table S3. An improvement in discrimination does not by itself imply an improvement in calibration, and both were therefore assessed: optimism-corrected calibration slopes were at least 0.98 throughout, and reweighting did not degrade calibration in any cohort and improved it in several instances.

The reweighted indices also showed superior discrimination for severe non-fatal complications in the validation cohorts (Tables 3 and 4). For severe bleeding (≥ 5 red blood cell concentrates), AUC decreased from 0.758 to 0.757 (Charlson) and increased from 0.826 to 0.862 (Elixhauser) in endometrial cancer; 0.703 to 0.730 and 0.787 to 0.851 in interventional left atrial appendage closure; and 0.712 to 0.739 and 0.839 to 0.900 in STEMI. For prolonged mechanical ventilation (> 48 h), the corresponding changes were from 0.776 to 0.786 (Charlson) and from 0.854 to 0.889 (Elixhauser) in endometrial cancer; from 0.671 to 0.734 and 0.806 to 0.862 in interventional left atrial appendage closure; and from 0.641 to 0.673 and 0.830 to 0.871 in STEMI.

**Table 3:** Model discrimination regarding the endpoint severe bleeding (transfusion of more than five red blood cell concentrates)

	External validation cohorts		
	Elective endometrial cancer	Interventional left atrial appendage closure	Emergency STEMI
	(*N* = 85,204)	(*N* = 28,039)	(*N* = 248,062)
	AUC [95% CI]	AUC [95% CI]	AUC [95% CI]
Charlson score	0.758 [0.743–0.772]	0.703 [0.683–0.723]	0.712 [0.705–0.718]
Charlson score - reweighted	0.757 [0.742–0.771]	0.730 [0.711–0.749]	0.739 [0.733–0.746]
Elixhauser score	0.826 [0.812–0.839]	0.787 [0.769–0.804]	0.839 [0.833–0.844]
Elixhauser score - reweighted	0.862 [0.849–0.875]	0.851 [0.836–0.866]	0.900 [0.896–0.905]

**Table 4:** Model discrimination regarding the endpoint prolonged mechanical ventilation (> 48 h)

	External validation cohorts		
	Elective endometrial cancer	Interventional left atrial appendage closure	Emergency STEMI
	(*N* = 85,204)	(*N* = 28,039)	(*N* = 248,062)
	AUC [95% CI]	AUC [95% CI]	AUC [95% CI]
Charlson score	0.776 [0.758–0.793]	0.671 [0.642-0.700]	0.641 [0.638–0.645]
Charlson score - reweighted	0.786 [0.768–0.805]	0.734 [0.706–0.762]	0.673 [0.669–0.677]
Elixhauser score	0.854 [0.838–0.870]	0.806 [0.783–0.830]	0.830 [0.827–0.832]
Elixhauser score - reweighted	0.889 [0.874–0.904]	0.862 [0.842–0.881]	0.871 [0.869–0.874]

Predictive performance for continuous outcomes (Tables 5 and 6) followed the same pattern. For total reimbursement, R² was higher for the reweighted Elixhauser compared to the original Elixhauser in two of three validation cohorts (endometrial cancer: 0.137 → 0.174; left atrial appendage closure: 0.038 → 0.057), while in STEMI the difference was negligible and confidence intervals overlapped substantially (0.135 [0.130–0.140] vs. 0.131 [0.126–0.135]). RMSE was modestly reduced in endometrial cancer and left atrial appendage closure, but marginally higher in STEMI. These improvements were statistically non-significant and should be interpreted with caution.

**Table 5:** Predictive performance measures (R², RMSE) regarding the endpoint total reimbursement

	External validation cohorts		
	Elective endometrial cancer	Interventional left atrial appendage closure	Emergency STEMI
	(*N* = 85,204)	(*N* = 28,039)	(*N* = 248,062)
	R^2^ [95% CI]	R^2^ [95% CI]	R^2^ [95% CI]
Charlson score	0.102 [0.086–0.119]	0.017 [0.013–0.022]	0.043 [0.041–0.046]
Charlson score - reweighted	0.100 [0.086–0.115]	0.026 [0.019–0.032]	0.061 [0.058–0.064]
Elixhauser score	0.137 [0.118–0.155]	0.038 [0.031–0.046]	0.135 [0.130–0.140]
Elixhauser score - reweighted	0.174 [0.156–0.193]	0.057 [0.048–0.065]	0.189 [0.182–0.196]
	RMSE [95% CI]	RMSE [95% CI]	RMSE [95% CI]
Charlson score	5525 [5039–6011]	5925 [5051–6799]	10,613 [10337–10888]
Charlson score - reweighted	5531 [5052–6011]	5899 [5026–6772]	10,513 [10234–10791]
Elixhauser score	5418 [4936–5900]	5860 [4991–6729]	10,094 [9813–10374]
Elixhauser score - reweighted	5299 [4829–5768]	5805 [4944–6666]	9769 [9485–10051]

**Table 6:** Predictive performance measures (R², RMSE) regarding the endpoint length of hospital stay

	External validation cohorts		
	Elective endometrial cancer	Interventional left atrial appendage closure	Emergency STEMI
	(*N* = 85,204)	(*N* = 28,039)	(*N* = 248,062)
	R^2^ [95% CI]	R^2^ [95% CI]	R^2^ [95% CI]
Charlson score	0.093 [0.086-0.100]	0.086 [0.078–0.094]	0.083 [0.080–0.086]
Charlson score - reweighted	0.104 [0.095–0.113]	0.124 [0.115–0.133]	0.096 [0.093-0.100]
Elixhauser score	0.124 [0.115–0.134]	0.162 [0.147–0.177]	0.155 [0.150–0.160]
Elixhauser score - reweighted	0.155 [0.145–0.165]	0.226 [0.212–0.240]	0.166 [0.162–0.171]
	RMSE [95% CI]	RMSE [95% CI]	RMSE [95% CI]
Charlson score	8.47 [8.21–8.72]	8.36 [7.97–8.74]	8.23 [8.07–8.39]
Charlson score - reweighted	8.42 [8.15–8.68]	8.18 [7.79–8.57]	8.17 [8.01–8.33]
Elixhauser score	8.32 [8.06–8.58]	8.00 [7.60–8.40]	7.90 [7.74–8.06]
Elixhauser score - reweighted	8.17 [7.92–8.43]	7.69 [7.32–8.07]	7.85 [7.69–8.01]

**Fig. 1:**
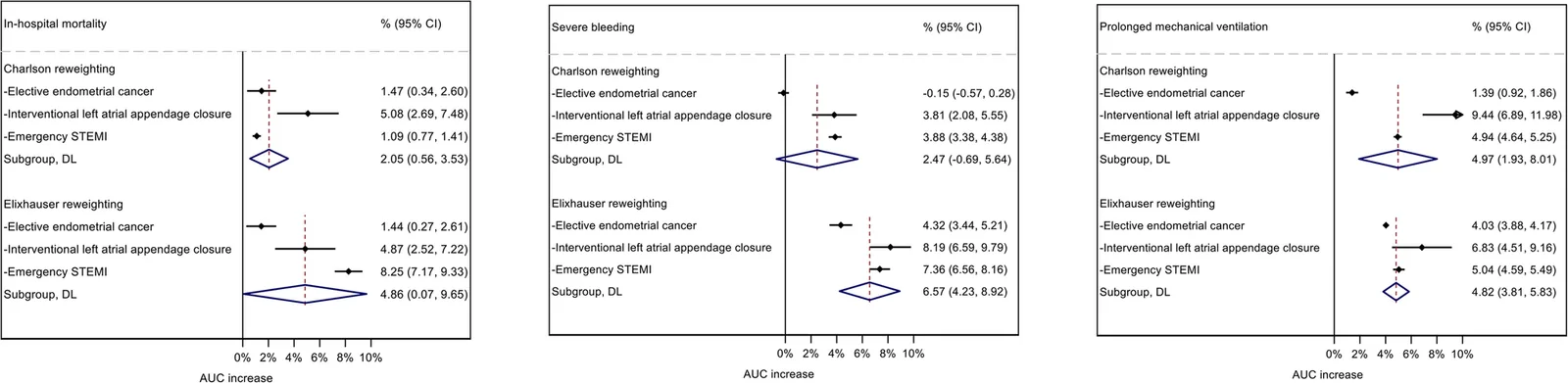
Relative AUC improvement after reweighting in the validation cohorts, random effects meta-analyses according to the DerSimonian-Laird model. AUC: area under the receiver operating characteristic curve; CI: confidence interval; DL: DerSimonian-Laird; STEMI: ST-elevation myocardial infarction

**Fig. 2:**
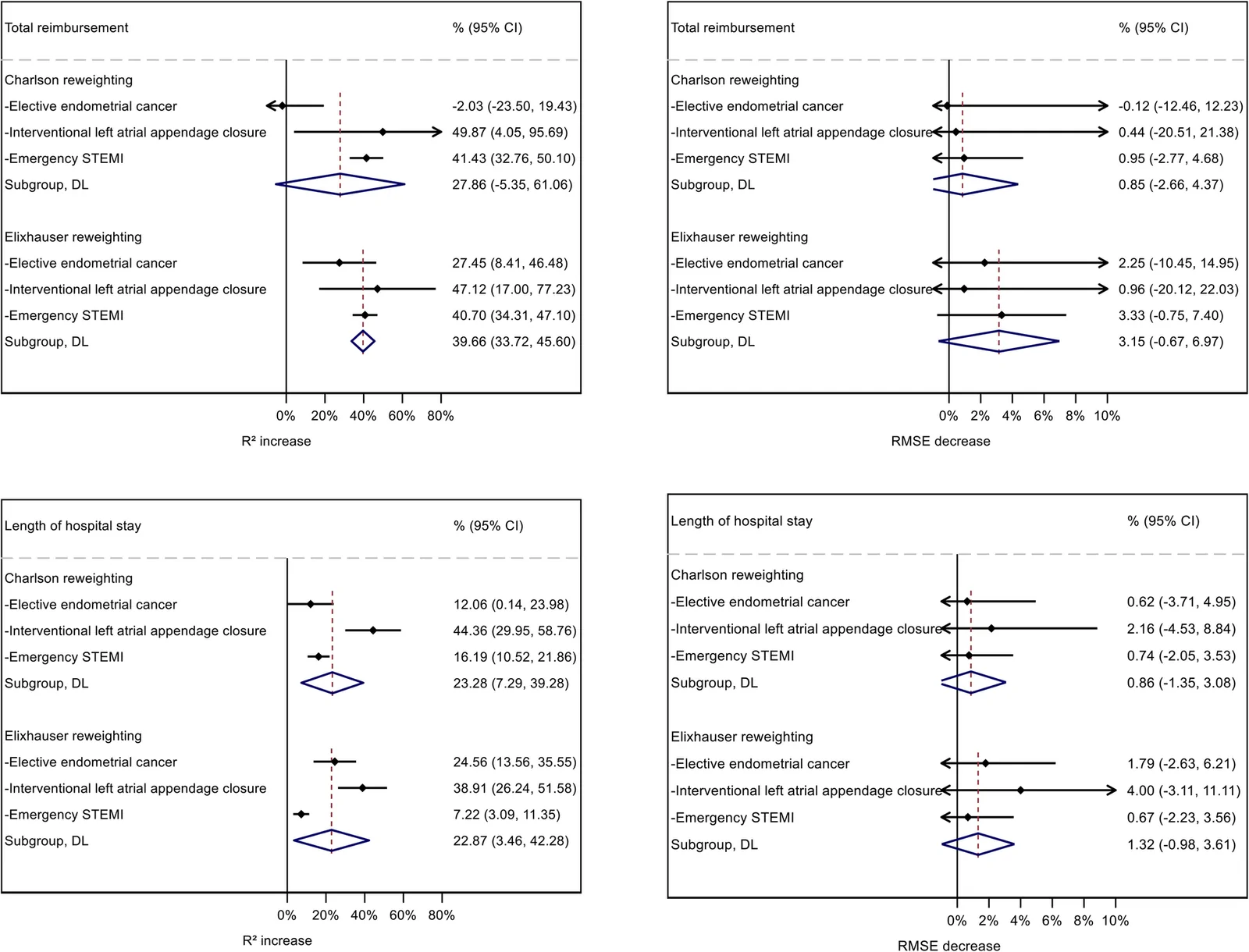
Relative improvement of R² and RMSE after reweighting in the validation cohorts, random effects meta-analyses according to the DerSimonian-Laird model. CI: confidence interval; DL: DerSimonian-Laird; RMSE: root mean square error; STEMI: ST-elevation myocardial infarction

Figure 1 presents forest plots of the meta-analyzed relative improvements in model performance across the three validation cohorts. For dichotomous clinical endpoints, the analyses demonstrated that reweighting consistently improved model discrimination. Reweighted Elixhauser models achieved a greater pooled relative AUC increase for in-hospital mortality (4.86% [0.07–9.65]) and severe bleeding (6.57% [4.23–8.92] compared to reweighted Charlson models (2.05% and 2.47%, respectively). For prolonged mechanical ventilation, both reweighted indices yielded comparable pooled improvements of approximately 5%. A similar pattern was observed for continuous outcomes (see Fig. 2), where reweighting enhanced explained variance (R²). The pooled relative R² increase for total reimbursement was again larger for the Elixhauser index (39.66% [33.72–45.60]) than for the Charlson index (27.86% [-5.35–61.06]). For length of hospital stay, both indices showed significant pooled R² increases of over 20%. In contrast, the corresponding pooled relative decreases in RMSE were modest and not statistically significant for either continuous outcome. Because only three validation cohorts contribute to each pooled estimate, these meta-analytic summaries are exploratory; the cohort-specific results in Tables 2, 3, 4, 5 and 6 remain the primary basis for interpretation, and the pooled values should be read as weighted summaries of these cohort-specific findings rather than as generalisable pooled effects.

## Discussion

Overall, the data-driven reweighting of established comorbidity indices improved discrimination for mortality and complications and modestly enhanced predictions for resource-related outcomes. The pattern of weights assigned to several Elixhauser conditions fits well with findings from earlier large-scale evaluations. Conditions such as advanced malignancy and coagulopathy received strong positive weights, while others like uncomplicated arterial hypertension were assigned negative weights. This is consistent with previous work, including the Swiss Elixhauser reweighting by Sharma et al., which also found higher C-statistics for Elixhauser based models compared with Charlson based models [8].

The observed AUC gains with adaptive lasso-based reweighting parallel findings from diagnoses-based reweighting in frailty research, where a reweighted Hospital Frailty Risk Score substantially improved discrimination and calibration relative to the original weighting [9]. Because discrimination and calibration are distinct properties of a risk-adjustment model, both were evaluated here; the quantitative metrics in Supplementary Table S3 indicate that the AUC gains obtained by reweighting were not achieved at the expense of calibration.

These findings add to the literature on comorbidity-based risk adjustment by jointly evaluating discrimination, explained variance, and prediction error across different endpoint types. Traditionally, the Charlson and Elixhauser comorbidity indices have been used as covariates for risk adjustment in observational health-economic and clinical outcomes studies, primarily assessed through discrimination measures such as the AUC [4–8]. Most prior validation efforts have focused exclusively on binary outcomes such as mortality or complications, with performance expressed in terms of AUC or C-statistics, and occasionally calibration [8, 9]. However, when comorbidity scores are used to explain variation in resource utilization (such as length of stay or reimbursement) variance-based metrics such as R² or error measures like the RMSE become more appropriate. Despite this, few studies in health economics or health-services research report these continuous-performance metrics alongside discrimination indices, and comparative analyses that integrate all three remain rare [20].

This analysis helps address a methodological gap by applying adaptive lasso reweighting to both indices and validating their performance across mortality, complication, and cost-related outcomes. This multi-endpoint approach highlights that the predictive validity of comorbidity weighting is not limited to survival or binary event prediction but extends meaningfully to economic outcomes, supporting its use in multifaceted health-economic evaluations. In other domains, authors have emphasized the limitations of relying on single performance criteria, advocating for joint reporting of error, variance, and discrimination metrics to enhance interpretability and comparability [21, 22]. Nevertheless, within health economics and machine learning for outcomes research, current reviews indicate that reporting remains fragmented. Recent reviews of machine learning applications in health economics and outcomes research [23, 24] found that most studies focus on regression-based performance (R², RMSE) for continuous cost predictions or AUC-based discrimination for binary risk stratification, but almost none systematically evaluate both within the same analytic framework. Even in more general predictive modeling contexts (such as prediction of insurance costs [25, 26] or outpatient wait times [27]) R² and RMSE are reported for regression tasks while AUC is confined to classification tasks, emphasizing the separation between modeling paradigms rather than their integration. As noted in the Methods, the meta-analytic pooling across three heterogeneous cohorts is exploratory; cohort-specific results in Tables 2, 3, 4, 5 and 6 remain the primary basis for interpretation.

The consistent improvements observed with reweighted scores across mortality, complication, and cost-related outcomes suggest that adaptive penalization improves the generalizability of comorbidity weights beyond their original disease-specific context. These findings support the use of the reweighted Charlson and Elixhauser indices as adjustment tools in observational effectiveness and health-economic analyses within the German claims-data setting examined here, and they indicate that discrimination-oriented and variance-oriented model evaluation can usefully be reported alongside each other.

In the present analysis, we focused the comparison mainly on the Charlson index weighted according to Deyo et al. (1992) [3] and the Elixhauser index weighted according to van Walraven et al. (2009) [6], as these are the most widely implemented adaptations in contemporary observational and administrative data research. Both sets of weights have become standard for risk adjustment in studies using hospital discharge data, owing to their empirical derivation and extensive validation across healthcare systems [Deyo et al. (1992) [3]; van Walraven et al. (2009) [6]. Alternative weighting schemes were also examined as part of our supplementary analyses, including the Charlson weights by Quan et al. (2005) [7], Schneeweiss et al. (2003) [4], and Bannay et al. (2016) [5], as well as the original Elixhauser weights [Elixhauser et al. (1998) [2] and the Swiss adaptation by Sharma et al. (2021) [8]. As shown in Table S2 of the supplementary appendix, none of these alternative versions achieved better discrimination for in-hospital mortality than the Deyo- or van Walraven-based implementations in the development cohort. For instance, AUC values for the Charlson score ranged from 0.728 (Quan) to 0.759 (Bannay), compared with 0.759 for Deyo and 0.786 for the reweighted version; similarly, for the Elixhauser score, AUC were 0.791 (original), 0.832 (van Walraven), and 0.833 (Sharma), while the reweighted index reached 0.865. Expressed as absolute incremental gains over the best-performing existing variant, the reweighted Elixhauser improved AUC by 0.033 over the van Walraven weights (0.865 vs. 0.832) and by 0.032 over the Swiss Elixhauser weights (0.865 vs. 0.833). For Charlson, the reweighted version exceeded the Deyo and Bannay variants by 0.027 (0.786 vs. 0.759). Although these gains are modest, they are consistent across all external validation cohorts. These findings confirm that, within this dataset, the more recent alternative weightings did not materially improve discrimination compared to the well-established Deyo and van Walraven systems. Accordingly, we limited discussion in the main text to these canonical reference versions, which remain the most appropriate benchmarks for assessing the incremental performance gains achieved through adaptive lasso reweighting.

From a practical health-economic perspective, improved risk adjustment with reweighted indices may, in principle, influence three classes of applied analysis. First, in observational treatment comparisons (e.g., comparing costs or outcomes across hospital or procedure types), more accurate baseline risk control may reduce residual confounding and thereby alter estimated treatment differentials. Second, in hospital benchmarking and quality reporting, better risk adjustment may reduce the likelihood of unfair performance attribution to hospitals treating more complex patients. Third, in budget impact and cost-effectiveness models that rely on comorbidity-stratified cost predictions, a higher R² may reduce uncertainty in cost projections. We emphasise that these three classes of application are hypotheses rather than demonstrated effects: the present study does not evaluate any downstream treatment comparison, benchmarking exercise, or budget-impact model, and the practical consequences of the observed performance gains therefore remain to be established empirically. While the magnitude of R² gains observed for total reimbursement (e.g., endometrial cancer: 0.137 → 0.174) is consistent with a potentially meaningful impact in studies where comorbidity burden is a major explanatory variable for resource use, confirming this will require dedicated applied analyses.

Several limitations apply, similar to those seen in studies based on administrative or claims data. First, as with similar reweighting efforts using hospital billing data [8, 9], the approach depends entirely on routinely coded ICD-10 diagnoses. These codes are assigned primarily for reimbursement rather than research, and coding intensity may vary across hospitals, specialties, and case mix. Consequently, certain comorbidities or complications might be underreported, particularly in emergency or short-stay admissions, where the full diagnostic spectrum is less likely to be documented. Second, as ICD-10 data typically lack temporal information, it is impossible to distinguish between pre-existing comorbidities present on admission and conditions developing during hospitalization. This limitation may lead to partial misclassification, particularly for conditions such as anemia, renal disease, or coagulopathy, which may represent both pre-existing risk factors and in-hospital complications. This concern is especially relevant for outcomes such as severe bleeding and prolonged mechanical ventilation, where some predictor diagnoses may partly reflect the index event rather than baseline morbidity. While restricting analyses to diagnoses from prior admissions would in principle address this issue, this was not feasible in the current dataset structure, and access to prior admission records was not available for the validation cohorts. We note, however, that this limitation applies equally to all established Charlson and Elixhauser weighting schemes used as comparators, and therefore does not systematically disadvantage the reweighted indices relative to existing approaches. Third, although our data include the full patient population of a tertiary care teaching hospital, the development cohort is single-center, and while external validation across independent cohorts mitigates this, some degree of limited generalizability to other health systems remains possible. Fourth, as in previous administrative-data studies, we were unable to assess clinical severity or laboratory parameters that might further refine risk adjustment. Fifth, a potential limitation of the reweighted indices is that several conditions (e.g., uncomplicated and complicated arterial hypertension, hypothyroidism, depression) receive negative weights, which may appear counterintuitive at the individual-patient level. In multivariable penalized regression, negative coefficients can arise from conditioning effects — patients coded with these conditions in the presence of other diagnoses may represent a clinically less severely ill subgroup — or from coding conventions in administrative data, where these diagnoses are more completely documented in lower-acuity planned admissions. This pattern is consistent with prior reweighting studies [8], including the van Walraven Elixhauser system [6], and does not indicate that these conditions are biologically protective. Users applying the reweighted indices in applied analyses should be aware that negative-weight conditions may produce counterintuitive score contributions in individual cases. Even where aggregate predictive performance improves, as demonstrated in the validation cohorts, negative weights complicate the interpretation and the communication of results, in particular for clinical or non-technical audiences, for whom a comorbidity that appears to “reduce” risk is difficult to convey. We therefore recommend that the weighting scheme be reported transparently whenever the reweighted indices are used, and that interpretation focus on aggregate model performance rather than on the contribution of individual conditions. Sixth, length of stay and total reimbursement have markedly right-skewed, heavy-tailed distributions, as is consistently reported for German DRG data. Linear regression with RMSE may therefore be disproportionately influenced by a small number of extreme cases, potentially yielding underestimates of predictive performance in the central range of the distribution; the variance-based R², by contrast, is less sensitive to this and remains informative. We were unable to report further distributional statistics (e.g., skewness or percentiles) for these outcomes, as the underlying patient-level data are held by the Federal Statistical Office and each additional aggregate query is subject to a separate statistical-disclosure review. The extent to which the heavy tails attenuate the continuous-outcome results can therefore not be quantified here, and the RMSE values in Tables 5 and 6 should be read as conservative indicators of predictive accuracy rather than as precise estimates of the prediction error in the central range of the distribution. Alternative modelling strategies (log-transformed outcomes, or generalised linear models with a gamma or negative-binomial family) may provide more robust estimates and are a topic for future work. Seventh, the validation cohorts span up to 13 years (2007–2020 for endometrial cancer). Over this period, German DRG reimbursement rates, coding intensity rules, and case-mix composition evolved substantially. Reimbursement figures were not adjusted for these temporal changes, which may have introduced heteroscedasticity and attenuated explanatory performance in the continuous-outcome analyses, particularly in the endometrial cancer cohort. Finally, coding practices and reimbursement incentives may systematically influence the recording of specific comorbidities, introducing potential bias in risk adjustment models based on claims data. Despite these limitations, routine administrative data remain essential for large-scale model development and comparative health-economic research, as long as their constraints are recognized and addressed through external validation and sensitivity checks.

## Supplementary Information

Supplementary Material 1.

## Data Availability

Data are available upon reasonable request. Regarding the validation cohorts, the patient data are stored on the server of the Federal Statistical Office of Germany and are not available due to data protection. The calculated raw data are sent anonymized to the scientists.
