# Supplementary material for: Data-driven reweighting of the Charlson and Elixhauser comorbidity indices using German claims data: development for in-hospital mortality only, external validation across clinical and health-economic outcomes
Source: Health Econ Rev. 2026 Oct 27;16:130. doi: 10.1186/s13561-026-00888-9 (PMC13721275; doi:10.1186/s13561-026-00888-9)
Supplement: Supplementary file 1 [file 13561_2026_888_MOESM1_ESM.docx]

**Table S1: Score development in the Freiburg cohort (all patients hospitalized between 2012 and 2015 in Freiburg, N=198,819)**

|  |  | **Charlson score** | | | | |  | **Elixhauser score** | | | |
| --- | --- | --- | --- | --- | --- | --- | --- | --- | --- | --- | --- |
| **Charlson Comorbidities** | **Mean** | **Deyo** | **Quan** | **Schneeweiss** | **Bannay** | **reweighted** |  | **Elixhauser** | **Walraven** | **Swiss** | **reweighted** |
| Myocardial infarction | 3.53% | 1 | 0 | 1 | 0 | 2 |  |  |  |  |  |
| Congestive heart failure | 3.85% | 1 | 2 | 1 | 2 | 4 |  | 1 | 7 | 13 | 2 |
| Peripheral vascular disease | 6.70% | 1 | 0 | 1 | 1 | 1 |  | 1 | 2 | 3 | 1 |
| Cerebrovascular disease | 8.06% | 1 | 0 | 1 | 1 | 4 |  |  |  |  |  |
| Dementia | 1.36% | 1 | 2 | 3 | 2 | 2 |  |  |  |  |  |
| Chronic pulmonary disease | 7.67% | 1 | 1 | 2 | 1 | 1 |  | 1 | 3 | 3 | 0 |
| Rheumatologic disease | 0.56% | 1 | 1 | 0 | 0 |  |  |  |  |  |  |
| Peptic ulcer disease | 0.54% | 1 | 0 | 0 | 0 | 4 |  | 1 | 0 | 0 | 2 |
| Mild liver disease | 3.25% | 1 | 2 | 2 | 2 | 2 |  | 1 | 11 | 16 | 1 |
| Diabetes without chronic complications | 10.41% | 1 | 0 | 1 | 0 |  |  | 1 | 0 | 1 | 0 |
| Diabetes with chronic complications | 2.17% | 2 | 1 | 2 | 0 | 0 |  | 1 | 0 | -1 |  |
| Hemiplegia or paraplegia | 4.84% | 2 | 2 | 1 | 2 | 2 |  | 1 | 7 | 11 | 3 |
| Renal disease | 8.65% | 2 | 1 | 3 | 1 | 2 |  | 1 | 5 | 8 | 1 |
| Any malignancy | 21.77% | 2 | 2 | 2 | 2 | 2 |  |  |  |  |  |
| Moderate or severe liver disease | 0.90% | 3 | 4 | 3 | 3 | 5 |  |  |  |  |  |
| Metastatic solid tumor | 10.36% | 6 | 6 | 6 | 11 | 4 |  | 1 | 12 | 17 | 3 |
| AIDS/HIV | 0.25% | 6 | 4 | 6 | 1 |  |  | 1 | 0 | 0 |  |
| **Additional Elixhauser Comorbidities** |  |  |  |  |  |  |  |  |  |  |  |
| Cardiac arrhythmias | 12.09% |  |  |  |  |  |  | 1 | 5 | 6 | 3 |
| Valvular disease | 2.75% |  |  |  |  |  |  | 1 | -1 | -1 |  |
| Pulmonary circulation disorders | 1.92% |  |  |  |  |  |  | 1 | 4 | 6 | 2 |
| Hypertension, uncomplicated | 31.65% |  |  |  |  |  |  | 1 | 0 | -4 | -1 |
| Hypertension, complicated | 1.35% |  |  |  |  |  |  | 1 | 0 | -3 | -2 |
| Other neurological disorders | 7.49% |  |  |  |  |  |  | 1 | 6 | 10 | 3 |
| Hypothyroidism | 7.43% |  |  |  |  |  |  | 1 | 0 | -3 | -1 |
| Lymphoma | 2.68% |  |  |  |  |  |  | 1 | 9 | 9 | 0 |
| Solid tumor without metastasis | 18.13% |  |  |  |  |  |  | 1 | 4 | 10 | 1 |
| Rheumatoid arthritis | 2.10% |  |  |  |  |  |  | 1 | 0 | -1 | 0 |
| Coagulopathy | 8.22% |  |  |  |  |  |  | 1 | 3 | 9 | 5 |
| Obesity | 6.33% |  |  |  |  |  |  | 1 | -4 | -6 | -1 |
| Weight loss | 2.15% |  |  |  |  |  |  | 1 | 6 | 6 | 3 |
| Fluid and electrolyte disorders | 11.16% |  |  |  |  |  |  | 1 | 5 | 5 | 4 |
| Blood loss anemia | 0.71% |  |  |  |  |  |  | 1 | -2 | -5 |  |
| Deficiency anemia | 1.54% |  |  |  |  |  |  | 1 | -2 | -7 | -1 |
| Alcohol abuse | 3.38% |  |  |  |  |  |  | 1 | 0 | -3 |  |
| Drug abuse | 5.45% |  |  |  |  |  |  | 1 | -7 | -5 | -1 |
| Psychoses | 0.46% |  |  |  |  |  |  | 1 | 0 | -4 |  |
| Depression | 5.43% |  |  |  |  |  |  | 1 | -3 | -3 | -1 |

**Table S2: Model discrimination regarding the endpoint in-hospital mortality**

|  | **Development cohort** |  |  |  |
| --- | --- | --- | --- | --- |
|  | **All patients in Freiburg** |  |  |  |
|  | (N=198,819) |  |  |  |
|  | **AUC [95%CI]** |  |  |  |
| Charlson score - Deyo | 0.759 [0.752-0.766] |  |  |  |
| Charlson score - Quan | 0.728 [0.720-0.736] |  |  |  |
| Charlson score - Schneeweiss | 0.752 [0.745-0.759] |  |  |  |
| Charlsonscore - Bannay | 0.759 [0.752-0.766] |  |  |  |
| **Charlson score - reweighted** | **0.786 [0.780-0.793]** |  |  |  |
| Elixhauser score - original | 0.791 [0.784-0.797] |  |  |  |
| Elixhauser score - Walraven | 0.832 [0.826-0.837] |  |  |  |
| Elixhauser score - Swiss | 0.833 [0.827-0.839] |  |  |  |
| **Elixhauser score - reweighting** | **0.865 [0.860-0.871]** |  |  |  |

**Figure S1: Calibration plots for in-hospital mortality in the three validation cohorts**

| **Elective endometrial cancer**  Charlson score   | Charlson score – reweighted   | Elixhauser score   | Elixhauser score – reweighted   |
| --- | --- | --- | --- |
| **Interventional left atrial appendage closure**  Charlson score   | Charlson score – reweighted   | Elixhauser score   | Elixhauser score – reweighted   |
| **Emergency ST-elevation myocardial infarction**  Charlson score   | Charlson score – reweighted   | Elixhauser score   | Elixhauser score – reweighted   |

**Quantitative calibration of the comorbidity indices (complementing Figure S1)**

Adequate risk adjustment requires both discrimination and calibration. To complement the flexible calibration plots in Figure S1, we quantified calibration for each index in each validation cohort using three metrics: calibration-in-the-large (CITL), the calibration slope, and the integrated calibration index (ICI). Within each cohort, a logistic regression of in-hospital mortality on the index was fitted and the predicted probability obtained for each patient. Patient-level outcomes were reconstructed from the mortality-by-index-level tabulations supplied by Destatis. Because the indices enter risk-adjustment models estimated within the same cohort, CITL is essentially zero and the apparent calibration slope is 1 by construction; we therefore report the heuristic, optimism-corrected calibration slope (Van Houwelingen/Copas shrinkage factor, (χ² − df)/χ²) to confirm that expected out-of-sample shrinkage is negligible. CITL is a property of the *setting* rather than of the *index*: with baseline mortality differing more than ten-fold across the three cohorts (≈0.8 % in endometrial cancer, ≈1.2–1.5 % in left atrial appendage closure, ≈11 % in STEMI), absolute predicted risks are not transportable between these populations without recalibration of the model intercept. The ICI — the patient-weighted mean absolute difference between observed and predicted mortality — is not constrained by construction and is therefore the substantive, discriminating measure of residual miscalibration, directly comparable between the original and the reweighted indices.

Calibration was excellent for every index in every cohort (Table S3). The optimism-corrected calibration slope was ≥ 0.98 throughout (most values ≈ 1.00), indicating negligible overfitting, and CITL was essentially zero within each cohort. The ICI was below 0.4 percentage points in the endometrial cancer and left atrial appendage closure cohorts and below 2.4 percentage points in the higher-risk STEMI cohort. Importantly, reweighting did not degrade calibration in any cohort and improved it in several instances — most clearly for the Elixhauser index in STEMI (ICI 2.40 % → 1.78 %) and for both indices in endometrial cancer (Charlson 0.34 % → 0.23 %; Elixhauser 0.35 % → 0.27 %). The largest residual miscalibration (original Elixhauser, STEMI) was thus the value most reduced by reweighting. These results indicate that the gains in discrimination obtained by reweighting were not achieved at the expense of calibration.

**Table S3. Within-cohort calibration of the comorbidity indices for in-hospital mortality.**

| Cohort | Index | N | Deaths | Baseline mortality | Calibration slope¹ | CITL² | ICI³ |
| --- | --- | --- | --- | --- | --- | --- | --- |
| **Endometrial cancer** | Charlson (original) | 85,204 | 660 | 0.77 % | 0.999 | ≈ 0.00 | 0.34 % |
|  | Charlson (reweighted) | 85,204 | 660 | 0.77 % | 0.999 | ≈ 0.00 | 0.23 % |
|  | Elixhauser (original) | 85,204 | 660 | 0.77 % | 0.999 | ≈ 0.00 | 0.35 % |
|  | Elixhauser (reweighted) | 85,204 | 660 | 0.77 % | 0.999 | ≈ 0.00 | 0.27 % |
| **STEMI** | Charlson (original) | 248,054 | 27,127 | 10.94 % | 1.000 | ≈ 0.00 | 0.51 % |
|  | Charlson (reweighted) | 248,055 | 27,126 | 10.94 % | 1.000 | ≈ 0.00 | 0.54 % |
|  | Elixhauser (original) | 237,742 | 26,962 | 11.34 % | 1.000 | ≈ 0.00 | 2.40 % |
|  | Elixhauser (reweighted) | 248,055 | 27,128 | 10.94 % | 1.000 | ≈ 0.00 | 1.78 % |
| **LAA closure** | Charlson (original) | 21,957 | 309 | 1.41 % | 0.982 | ≈ 0.00 | 0.14 % |
|  | Charlson (reweighted) | 20,248 | 305 | 1.51 % | 0.990 | ≈ 0.00 | 0.15 % |
|  | Elixhauser (original) | 27,716 | 324 | 1.17 % | 0.997 | ≈ 0.00 | 0.37 % |
|  | Elixhauser (reweighted) | 27,384 | 320 | 1.17 % | 0.998 | ≈ 0.00 | 0.35 % |

¹ Heuristic, optimism-corrected calibration slope (Van Houwelingen/Copas shrinkage factor, (χ² − df)/χ²). ² Calibration-in-the-large, assessed within each cohort; absolute values < 10⁻⁷. ³ Integrated calibration index: patient-weighted mean absolute difference between observed and predicted mortality. Small between-index differences in N reflect the number of patients with a calculable index value in the respective tabulation.
